# Radiation-induced white matter dysfunction in patients with nasopharyngeal carcinoma

**DOI:** 10.3389/fnins.2025.1548744

**Published:** 2025-03-10

**Authors:** Xingyou Zheng, Li Li, Jian-ming Gao, Yang Hu, Limeng Deng, Ya-fei Kang, Youming Zhang

**Affiliations:** ^1^Department of Medical Imaging, The Fourth Hospital of Changsha (Integrated Traditional Chinese and Western Medicine Hospital of Changsha, Changsha Hospital of Hunan Normal University), Changsha, Hunan, China; ^2^State Key Laboratory of Oncology in South China, Collaborative Innovation Center for Cancer Medicine, Sun Yat-sen University Cancer Center, Guangzhou, China; ^3^State Key Laboratory of Oncology in South China, Department of Radiation Oncology, Collaborative Innovation Center for Cancer Medicine, Sun Yat-sen University Cancer Center, Guangzhou, China; ^4^Independent Researcher, Shanghai, China; ^5^School of Information, Xi’an University of Finance and Economics, Xi’an, Shaanxi, China; ^6^Department of Radiology, Xiangya Hospital, Central South University, Changsha, China; ^7^National Clinical Research Center for Geriatric Diseases, Xiangya Hospital, Central South University, Changsha, Hunan, China

**Keywords:** nasopharyngeal carcinoma, radiation-induced brain injury, white matter, functional MRI (fMRI), visual cortex

## Abstract

Radiation-induced structural abnormalities in white matter (WM) have been reported in patients with nasopharyngeal carcinoma (NPC); however, the alterations in functional domain were insufficiently investigated. A total of 111 NPC patients were included and these patients, based on whether completed radiation therapy (RT) or not, were divided into pre-RT (*n* = 47) and post-RT (*n* = 64) groups. Functional connectivity strength (FCS) between WM regions (WW-FCS) and between WM and gray matter (GM) regions (GW-FCS) was used to investigate the radiation-induced changes in WM function. Compared with the pre-RT patients, post-RT NPC patients showed decreased WW-FCS in the left superior cerebellar peduncle, right anterior limb of internal capsule, bilateral posterior thalamic radiation, and left tapetum. Compared with the pre-RT patients, post-RT NPC patients showed decreased GW-FCS in the left caudate, bilateral visual cortex, and the right ventral prefrontal cortex. In the post-RT group, the GW-FCS in left visual cortex was negatively correlated with radiation dosage for the brain stem (r = -0.35, *p* = 0.039), and for the left temporal lobe (r = -0.46, *p* = 0.0058). The GW-FCS in right visual cortex was negatively correlated with radiation dosage for the left temporal lobe (r = –0.38, *p* = 0.025). Our findings of decreased WW-FCS and GW-FCS in such brain regions (such as visual cortex, posterior thalamic radiation, and anterior limb of internal capsule, as well as superior cerebellar peduncle) suggest potential functional impairments in visual and motor systems.

## Introduction

Nasopharyngeal carcinoma (NPC), particularly prevalent in east and southeast Asia, is a malignancy originating from the nasopharyngeal epithelium (Chen et al., 2019). While advances in radiation therapy (RT) and chemotherapy have greatly improved patient survival (Zhang et al., 2018; Chua et al., 2016), these treatments can also result in serious complications. Radiation-induced brain necrosis (RIBN) is especially concerning due to its severe clinical symptoms (Turnquist et al., 2020; Rube et al., 2023; Huang et al., 2020), including seizures, cognitive decline, and psychological disorders, which significantly impact survivors’ quality of life (Tang et al., 2012). Therefore, investigating the neural mechanisms of radiation-induced brain injury before the onset of RIBN is essential for effective clinical management.

Advanced neuroimaging techniques enable detailed *in vivo* assessment of radiation-induced changes in both brain structure and function. Using voxel-based morphology (VBM) and surface-based morphology (SBM), several structural MRI studies have reported reductions in gray matter volume, cortical thickness, and cortical surface area in the bilateral temporal lobes and regions associated with the default mode network (DMN), such as the inferior parietal lobules (Zhang et al., 2018; Lv et al., 2014; Lin et al., 2017). Such findings are in good agreement with functional MRI studies that have shown decreased regional homogeneity (ReHo) and/or fractional amplitude of low-frequency fluctuation (fALFF) in brain regions both within the radiation field (such as the medial and lateral temporal lobes) and outside of it (such as the posterior corpus callosum/precuneus and inferior parietal lobule) (Zhang et al., 2021; Zhang et al., 2020; Ding et al., 2018). These studies examine structural and functional changes in gray matter, contributing to uncovering the pathogenesis of radiation-induced brain injury, though they largely overlook the impact of radiation on structural integrity of white matter.

Diffusion tensor imaging (DTI) is a robust method for examining microstructural alterations in the human brain *in vivo*. Using tract-based spatial statistics (TBSS), recent DTI studies have examined radiation-induced abnormalities in structural connectivity, showing that, compared with pre-RT NPC patients, post-RT patients exhibited significantly lower fractional anisotropy (FA) values in the right frontal and parietal lobes, as well as higher mean diffusivity (MD) values in the right occipital lobe (Duan et al., 2016). Another WM structural network study (Chen et al., 2020) found that post-RT NPC patients exhibited reduced global properties in DMN and executive control network during the early-delayed period, while in the late-delayed period, nodal alterations occurred in the limbic system, indicating dynamic changes and reorganization of WM networks over time and providing insights into the evolving mechanisms of radiation-induced brain injury.

While these studies were conducted primarily in the structural domain, far less attention has been paid to functional alterations of the axonal connectivity in patients with NPC. There are increasing evidence implicating the neurobiological significance of low-frequency blood oxygen level-dependent (BOLD) fluctuations (LFBFs) in white matter (WM) (van Osch et al., 2009; Ding et al., 2013; Aslan et al., 2011). For example, specific subregions of the corpus callosum can be exclusively activated by tasks related to their functional roles (Courtemanche et al., 2018; Fabri et al., 2014; Gawryluk et al., 2011). Even in the resting state, the BOLD signal in WM displays a structured organization rather than random noise, and the WM function can also be modulated by various tasks (Ji et al., 2017). Interestingly, anatomical fiber bundles tracked through DTI data can be identified using resting-state fMRI as well (Marussich et al., 2017; Peer et al., 2017). These findings suggest that WM function, as detected by the BOLD signal, may offer a new perspective for unraveling the neural mechanisms underlying various brain disorders. Since prolonged radiation exposure can trigger pathological events like WM demyelination, oligodendrocyte and astrocyte damage or death (Monje and Palmer, 2003; Yang et al., 2017), investigating functional changes in WM may be crucial for understanding the early clinical phenotypes of radiation-induced brain injury.

Capitalizing on the comprehensive neuroimaging data (including high resolution T1 imaging data and resting-stage fMRI data) of patients with NPC, this study aimed to examine the WM region-wise functional connectivity (FC) pattern. Based on existing literature on WM structural changes in patients with NPC following RT (Duan et al., 2016; Chen et al., 2020) and considering that radiation-induced brain injury affects the brain as a whole (Yang et al., 2017), we hypothesized that: (1) The changes in region-wise WM function measured by functional connectivity strength (FCS) would be prominent in patients with NPC following RT; (2) Patients with NPC undergoing RT would exhibit widespread alterations in WM function, not limited to regions directly exposed to radiation (such as arcuate fasciculus, inferior longitudinal fasciculus in temporal lobes) but extending to distant brain areas (such as internal capsule and optic radiations); (3) The changes in FCS of WM regions would be negatively correlated with radiation dosage of temporal lobes.

## Materials and methods

### Subjects

This study included a total of 111 NPC patients, with 47 in the pre-RT group and 64 in the post-RT group, consisting of entirely distinct individuals. The nasopharyngeal lesions were staged from T1N0M0 to T4N3M0 in the pre-RT group, and T1N1M0 to T4N3M0 in the post-RT groups. All patients in pot-RT group underwent both Intensity-modulated radiation therapy (IMRT) and traditional two-dimensional radiotherapy (2D-CRT). For a comprehensive understanding of the specific RT protocols, one can refer to our earlier publications (Zhang et al., 2018). In cases of patients with stages IIb to IVa–b, the concurrent administration of chemoradiotherapy, optionally combined with neoadjuvant or adjuvant chemotherapy, was advised for NPC patients. This treatment was typically initiated 1–3 months prior to or following RT, utilizing one or more chemotherapeutic drugs, including cisplatin, nedaplatin, paclitaxel, and fluorouracil. The study received approval from our hospital’s Medical Research Ethics Committee, and all participants provided their written informed consent.

### MRI acquisition

Magnetic resonance imaging data was acquired on a 3.0 T Siemens TrioTim scanner. T1-weighted structural images were collected using an MPRAGE gradient echo sequence (echo time = 2.52 ms, repetition time = 1,900 ms, inversion time = 900 ms, flip angle = 9°, field of view = 256 mm^2^ × 256 mm^2^, matrix = 256 × 256, voxel size = 1 mm^3^ × 1 mm^3^ × 1 mm^3^, 176 sagittal slices). Resting-state BOLD images were collected with a gradient echo planar imaging sequence (repetition time = 2,400 ms, echo time = 30 ms, flip angle = 90°, field of view = 230 mm^2^ × 230 mm^2^, matrix = 64 × 64, voxel size = 3.59 mm^3^ × 3.59 mm^3^ × 4.32 mm^3^, 40 axial slices, and 240 volumes). Participants were informed to lie still with their eyes closed and remain awake.

### Image preprocessing and quality control

MRI data were pre-processed using FreeSurfer (v6.0) (Dale et al., 1999; Fischl et al., 1999; Greve and Fischl, 2009), AFNI (v20.0.19) (Cox, 1996; Taylor and Saad, 2013), ANTs (v2.2.0) (Tustison et al., 2021), and PhiPipe (v1.2) (Hu et al., 2023). The T1-weighted images were first processed using FreeSurfer’s recon-all routine, which mainly included bias-field correction, skull removal, tissue segmentation, and surface reconstruction procedures. Then the brain images were non-linearly registered into the MNI152 template using ANTs. For quality control, the raw data quality, brain extraction, tissue segmentation, and spatial registration results were visually checked to exclude anatomical abnormality, apparent artifacts and processing failures. The Euler number generated by FreeSurfer was used as a quantitative measure of raw data quality (Rosen et al., 2018). The Euler numbers of all subjects were converted into Modified Z-scores, and the subjects with a Modified Z-score lower than -3.5 were treated as outlier subjects and excluded from downstream analysis.

For BOLD fMRI data, the main processing steps consisted of: (1) removing the first five volumes; (2) head motion correction. Power’s frame-wise displacement (FD) metric was calculated to quantify head motion; (3) slice timing correction; (4) the median volume was rigidly registered into the corresponding T1-weighted brain image; (4) motion censoring. Volumes with FD > 0.5 mm were treated as motion outliers and interpolated with neighboring volumes; (5) nuisance regression, in which Friston’s 24-parameter head motion model, linear and quadratic trends were regressed out; (6) high-pass temporal filtering at 0.01 Hz; (7) the processed fMRI data were transformed into the MNI152 space based on the fMRI-T1 and T1-MNI152 registration results with a final isotropic resolution of 3 mm. These normalized fMRI data were used for functional connectivity (FC) analysis. For quality control, the raw data quality and spatial registration results were visually checked to exclude apparent artifacts and processing failures. Subjects with mean FD > 0.5 mm and outlier ratio > 0.3 (the ratio between motion outliers and total volumes) were also excluded.

### Functional connectivity analysis

A brain parcelation mask in the MNI152 space including 114 gray matter (GM) regions (100 cortical regions + 14 subcortical structures) and 50 white matter (WM) regions were created by combining the Schaefer 100-parcel atlas (Schaefer et al., 2018), Aseg atlas (Fischl et al., 2002) and JHU ICBM-DTI-81 WM atlas (Mori et al., 2008). The parcelation mask was applied to each participant to extract the mean time series of each brain region and the (Fisher’s Z transformed) Pearson correlation coefficient between the time series of any two regions was further calculated to obtain a 164 × 164 FC matrix. Based on the FC matrix, we further calculated two measures to reflect the region-wise FC pattern within the WM, and between GM and WM: (1) for each WM region, we calculated its average FC with other WM regions to reflect the functional connectivity strength (FCS) of this WM region with all other WM regions. This measure hereafter would be referred to as WW-FCS, (2) for each GM region, we calculated its average FC with WM regions to reflect the FCS of this GM region with all WM regions. This measure would be referred to as GW-FCS. The reason we focused on region-wise FCS measures instead of the FC between two regions is that we aimed to identify the loci most affected by radiation.

### Statistical analysis

To examine the WW-FCS and GW-FCS difference between pre-RTand post-RT groups, a multiple linear regression model was applied for each region. Age, sex and mean FD were included as covariates in the model. The multiple comparison correction was conducted using an FDR approach (Kim et al., 2008) and the significance level was set at an FDR-corrected *p*-value < 0.05. For WM and GM regions showing significant group differences, the associations between the maximum dosage of RT (MDRT) for the brainstem and temporal lobe and FCS were examined using a partial correlation analysis. Age, sex and mean FD were included as covariates. Of note, only 37 subjects of the post-RT group had the MDRT data. The statistical analysis was performed in the R environment (v4.4.1) and using the ppcor package (v1.0) (Kim, 2015). To test the statistical power of these analyses, we further conducted *post hoc* power analyses for the intergroup differences in WW-FCS and GW-FCS between pre-RT and post-RT groups, as well as for the partial correlation analyses between GW-FCS and MDRT.

## Results

### Demographic data and clinical parameters

The demographic and clinical data are displayed in Table 1. No significant differences were observed between the pre-RT and post-RT groups in age (*p* = 0.081), sex (*p* = 0.87), clinical staging (*p* = 0.675) and mean FD (*p* = 0.92).

**TABLE 1:** Demographic date and clinical parameters.

Clinical parameters	pre-RT group (*n* = 47)	post-RT group (*n* = 64)	*P*-value
**Age (years), mean (SD)**	46.70 (9.55)	43.39 (9.93)	0.081
**Gender, *n***			
Male	36	47	0.87
Female	11	17	
**Clinical staging (UICC/AJCC2009)**			
I/II	4^a^	7^b^	0.675
III/IV	34^a^	45^b^	
**Mean FD**	0.16 (0.067)	0.16 (0.076)	0.92

^a,b^Denotes 9 and 12 patients’ clinical staging were not available from medical records, respectively. Data in parentheses are SD. RT, radiation therapy; SD, standard deviation; UICC, International Union against Cancer; AJCC, American Joint Committee on Cancer.

### Decreased functional connectivity strength (FCS) in post-RT patients

**FIGURE 1:**
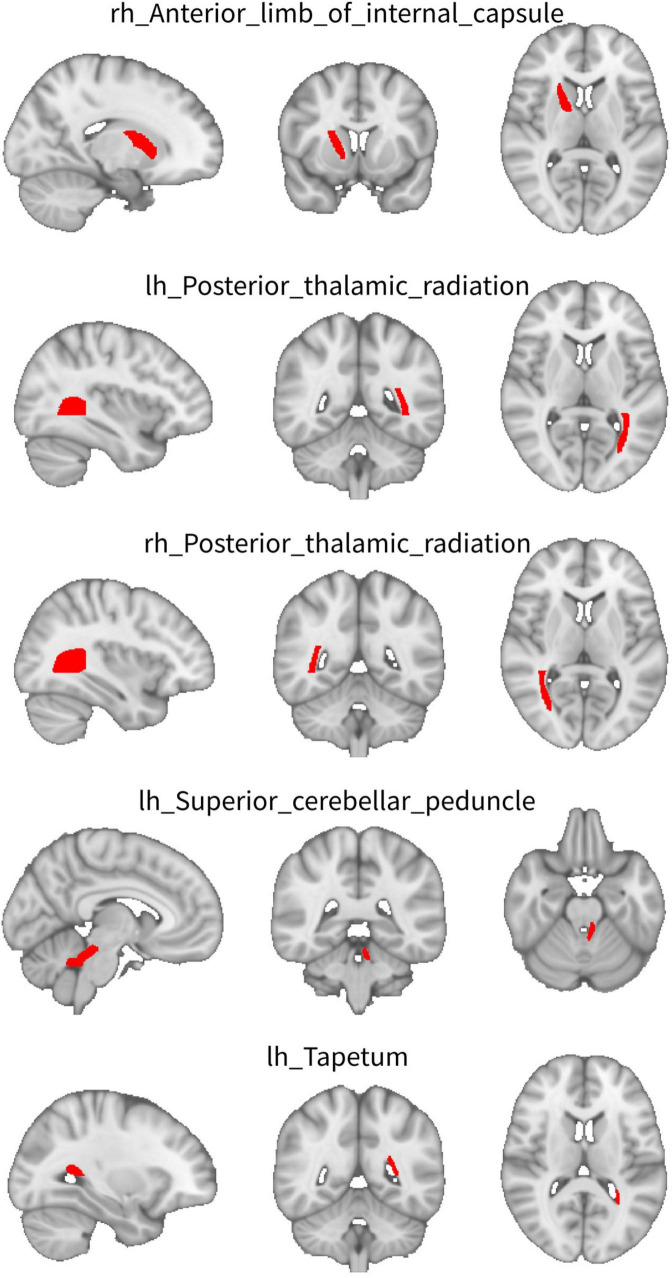
Between-group differences in functional connectivity strength (FCS) between WM regions (WW-FCS). Compared with pre-radiation therapy (RT) group, post-RT patients exhibited significantly decreased functional connectivity strength (FCS) in five white matter (WM) regions, including the left superior cerebellar peduncle, right anterior limb of internal capsule, right posterior thalamic radiation, left posterior thalamic radiation, and left tapetum.

For WW-FCS, compared with pre-RT group, patients in the post-RT group exhibited significantly decreased FCS in five WM regions, including the left superior cerebellar peduncle, right anterior limb of internal capsule, right posterior thalamic radiation, left posterior thalamic radiation, and left tapetum (Figure 1 and Table 2).

**TABLE 2:** Between-group differences in functional connectivity strength (FCS) between WM regions (WW-FCS) and between WM and gray matter (GM) regions (GW-FCS).

Brain region	pre-RT^1^	post-RT^1^	T-value	*P*-value	P-FDR_corr_
**WW-FCS**					
lh_Superior_cerebellar_peduncle	0.25 (0.11)	0.19 (0.10)	–2.99	0.0035	0.035
rh_Anterior_limb_of_internal_capsule	0.40 (0.13)	0.34 (0.11)	–3.19	0.0018	0.031
rh_Posterior_thalamic_radiation	0.39 (0.12)	0.31 (0.11)	–3.66	0.00039	0.020
lh_Posterior_thalamic_radiation	0.40 (0.13)	0.32 (0.11)	–3.32	0.0012	0.031
lh_Tapetum	0.22 (0.11)	0.16 (0.10)	–3.01	0.0033	0.035
**GW-FCS**					
rh_Caudate	0.43 (0.12)	0.36 (0.12)	–3.31	0.0013	0.043
lh_Vis_4	0.29 (0.10)	0.22 (0.11)	–3.26	0.0015	0.043
lh_Vis_5	0.32 (0.11)	0.26 (0.10)	–3.12	0.0023	0.045
lh_Vis_8	0.30 (0.12)	0.22 (0.11)	–3.42	0.00088	0.043
rh_Vis_4	0.31 (0.10)	0.25 (0.11)	–3.14	0.0022	0.045
rh_Default_PFCv_2	0.32 (0.15)	0.24 (0.13)	–3.33	0.0012	0.043

^1^Mean (standard deviation) for each group

**FIGURE 2:**
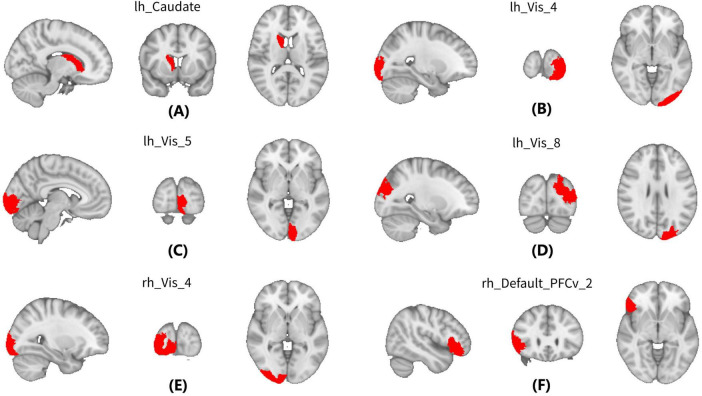
Between-group differences in between WM and gray matter (GM) regions (GW-FCS). Compared with Pre-radiation therapy (RT) group, patients in the post-RT group exhibited significantly decreased functional connectivity strength (FCS) in six gray matter (GM) regions, including left caudate (lh_Caudate) **(A)**, left visual cortex (lh_Vis_4, lh_Vis_5, and lh_Vis_8) **(B–D)**, right visual cortex subregion 4 (rh_Vis_4) **(E)**, and right ventral prefrontal cortex (rh_PFCv_2) **(F)**.

For GW-FCS, compared with pre-RT group, patients in the post-RT group exhibited significantly decreased FCS in six GM regions, including left caudate (lh_Caudate), left visual cortex (lh_Vis_4, lh_Vis_5, and lh_Vis_8), right visual cortex subregion 4 (rh_Vis_4), and right ventral prefrontal cortex (rh_PFCv_2) (Figure 2 and Table 2).

### GW-FCS was negatively associated with radiation dosage

**FIGURE 3:**
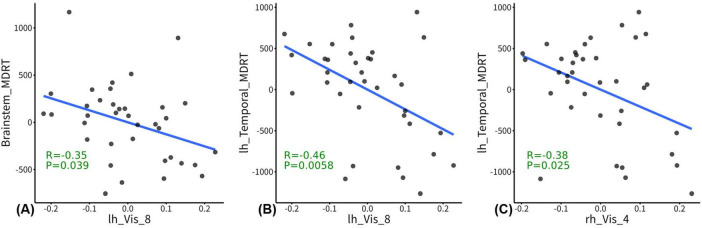
Correlations between functional connectivity strength (FCS) and radiation dosage. In the post-radiation therapy (RT) group, the between WM and gray matter (GM) regions (GW-FCS) in left visual cortex subregion 8 (lh_Vis_8) was negatively associated with maximum dosage of RT (MDRT) for the brain stem (*p* = 0.039), and MDRT for the left temporal lobe (*p* = 0.0058) **(A,B)**. In the post-RT group, the GW-FCS in right visual cortex subregion 4 (rh_Vis_4) was negatively associated with MDRT for the left temporal lobe (*p* = 0.025) **(C)**. The residuals of radiation dosage and GW-FCS after regressing out the covariates were used for visualization.

In the post-RT group, the GW-FCS in left visual cortex subregion 8 (lh_Vis_8) was negatively associated with MDRT for the brain stem (r = -0.35, *p* = 0.039), and MDRT for the left temporal lobe (r = -0.46, *p* = 0.0058) (Figures 3A, B). In the post-RT group, the GW-FCS in right visual cortex subregion 4 (rh_Vis_4) was negatively associated with MDRT for the left temporal lobe (r = -0.38, *p* = 0.025) (Figure 3C).

In the post-RT group, no significant correlations were observed between the MDRT and the WW-FCS of the five WM regions showing intergroup differences.

The results of the *post hoc* power analyses for the between-group differences in WW-FCS and GW-FCS, as well as the partial correlation analyses between GW-FCS and MDRT, are presented in Supplementary Tables 1, 2.

## Discussion

This is the first study to evaluate white matter functionality by analyzing functional connectivity strength, specifically investigating the effects of radiotherapy (RT) on the normal-appearing white matter (NAWM) in patients with NPC. We found decreased WW-FCS in the left superior cerebellar peduncle, right anterior limb of internal capsule, right posterior thalamic radiation, left posterior thalamic radiation, and left tapetum in post-RT patients compared with the pre-RT patients. Meanwhile, we also found decreased GW-FCS in brain regions (including left caudate, visual cortex, and right ventral prefrontal cortex). More interestingly, the GW-FCS in bilateral visual cortex were negatively associated with radiation dosage in the left temporal lobe or brainstem. These findings suggested potential radiation-induced disrupted in WM function in the integration of neural networks (such as visual network, motor and coordination network) in patients with NPC.

Our findings of the decreased WW-FCS in bilateral posterior thalamic radiation and GW-FCS in left visual cortex were consistent with previous neuroimaging studies, which reported decreased FA values and increased MD values in occipital white matter in post-RT NPC patients and reduced regional homogeneity (ReHo) values in the visual cortex in NPC patients with radiation-induced brain necrosis (Zhang et al., 2020; Duan et al., 2016). The thalamic radiation and visual cortex involve almost all key hubs of the visual network and have key roles in interpreting and interacting with the surrounding settings such as visual perception, object recognition, navigation and learning (Swienton and Thomas, 2014). Hence, impairments of visual pathway have been associated with a host of visual deficits or neurological disorders. Indeed, radiation-induced optic neuropathy is a common and devastating late complication in patients with NPC (Bodewitz and Keeren, 2011). Hu et al. (2003) reported a significant prolongation in visual evoked potential (VEP) latency, primarily occurring 1–2 years after radiotherapy in NPC patients, suggesting damage to the optic pathway. Given that the optic nerve, optic chiasm, and optic tract are located at the skull base and near the medial temporal lobe, these structures receive high doses of radiation during radiotherapy for NPC. We speculate that the posterior visual pathway WM dysfunction observed in this study may be a secondary effect of radiation-induced damage to these structures in anterior optic path. Such a speculation is further supported by our findings of significant negative correlations between the GW-FCS in bilateral visual cortex and radiation dosage in temporal lobe or brainstem.

Compared with pre-RT NPC patients, post-RT NPC patients exhibited reduced WW-FCS in the right anterior limb of the internal capsule and the left superior cerebellar peduncle, along with decreased GW-FCS in the right ventral prefrontal cortex. These results are in good agreement with previous functional and structural MRI studies investigating radiation-induced brain injury (Zhang et al., 2020; Ma et al., 2017). For example, using fALFF and FC, two fMRI studies reported altered local brain activities in cerebellum and bilateral frontal lobes as well as disrupted FC between cerebellum lobule VI and dorsolateral prefrontal cortex (Zhang et al., 2020; Ding et al., 2018; Ma et al., 2017). Anatomically, the ventral prefrontal cortex, the anterior limb of the internal capsule, and the contralateral superior cerebellar peduncle could form the cortico-pontine-cerebellar-cortical circuit, which is primarily responsible for the coordination and planning of movement (Ishihara et al., 1999). It is tempted to speculate that the functional abnormalities in such regions would result in motor control deficits, impaired coordination, and difficulties in executing physical activities. Such a speculation is further supported by previous neuroimaging and clinical studies, which reported that the motor dysfunction was common clinical symptom as evidenced by the observed reduced cortical thickness in precentral gyrus and decreased surface-based fALFF in paracentral lobule in NPC patients following RT (Lin et al., 2017; Zhang et al., 2021). The neural mechanisms underlying this phenomenon remain unclear. It is likely that the brainstem (including the pons) and the adjacent cerebellar peduncles, being close to the nasopharyngeal region, are within the radiation field and inevitably receive high-dose radiation exposure. Since the pons and cerebellar peduncles serve as relay stations in the cortico-pontine-cerebellar-cortical circuit, radiation damage to these areas may trigger functional abnormalities in the upstream pathways (including the frontopontine tract/anterior limb of internal capsule, and frontal cortex) or downstream pathways.

Our finding of decreased GW FCS in the left caudate is of particular interest. The functional significance of such result was twofold. First, the caudate (known as the neostriatum), together with its underlying axonal connectivity (such as internal capsule) constitute a neural pathway that functions in coordinating voluntary movement. As such, the functional differences that we observed in this region suggest that motor system is impaired in patients with radiation-induced brain injury. Such a speculation is supported by a previous subcortical structural MRI study showing significant atrophy in the left caudate in patients with NPC following concomitant chemoradiotherapy (Nan et al., 2022). Second, the caudate nuclei are also reported to be critical for the intentional control of behavior and thought (Villablanca, 2010). Two reported cases of selective infarctions or ischemia to bilateral caudate nuclei showed distractibility, aloofness and prospective memory decline (Narumoto et al., 2005; Canavero and Fontanella, 1998). It is likely that the observed abnormal GW FCS in the left caudate may contribute to the non-motor clinical complaints such as psychiatric disturbances and cognitive deficits in patients with NPC following RT. However, the exact neural mechanism that leads to these significantly GW functional alterations of caudate remains unclear. It is possible that multiple mechanisms, including inflammatory response and oxidative stress, are involved in the development of the brain injury after RT (Yang et al., 2017).

It was unexpected that there were no notable changes in WW-FCS or GW-FCS in brain areas subjected to intense radiation, such as the mesial temporal lobe and temporal pole. Such results suggest that radiation-induced white matter functional alterations in the inferior part of the temporal lobes are subtle and can be suppressed by more powerful alterations in the abovementioned neural networks (such as visual network, motor and coordination network). However, the exact cause of such an unexpected finding remains unknown. Off-target effects of RT on near-end brain regions (such as the mesial temporal lobe and temporal pole) that alter brain activity in far-end regions (such as caudate, internal capsule and ventral prefrontal cortex) may be one explanation (Ding et al., 2018; Beera et al., 2018). Our observations of significant alterations in WW-FCS or GW-FCS within the brain regions that form the visual network, as well as the motor and coordination network, indicate that future research should focus more on the functional and/or structural changes in distant brain areas, rather than those directly within the irradiated areas.

Several pathophysiological processes, including demyelination, inflammation, oxidative stress, and vascular injury, may contribute to radiation-induced white matter damage (Yang et al., 2017; Hwang et al., 2006). First, radiotherapy can directly damage oligodendrocytes, leading to demyelination and disrupted white matter integrity, which may impair neural signal transmission (Hwang et al., 2006; Lee et al., 2012) and contribute to decreased functional connectivity strength (WW-FCS and GW-FCS) in regions like the thalamic radiation, internal capsule, and cerebellar peduncles. Second, radiation-induced inflammation, driven by upregulated pro-inflammatory cytokines (e.g., TNF-α, IL-6) and microglial activation (Yang et al., 2017; Hwang et al., 2006; Raju et al., 2000), exacerbates demyelination and axonal damage. In addition, oxidative stress, caused by reactive oxygen species (ROS), overwhelms antioxidant defenses, resulting in lipid peroxidation, DNA damage, and apoptosis (Yang et al., 2017; Ilnytskyy and Kovalchuk, 2011; Koturbash et al., 2011), which is particularly harmful to metabolically active white matter. Furthermore, radiation-induced endothelial injury and blood-brain barrier disruption can lead to vascular leakage, ischemia, and secondary white matter degeneration (Ljubimova et al., 1991; Remler et al., 1986; Nonoguchi et al., 2011). These mechanisms likely interact synergistically, disrupting neural network integration and function (Yang et al., 2017). Therefore, future studies should further explore these pathways using multimodal imaging and biomarker analysis to better understand radiation-induced white matter injury and identify neuroprotective interventions.

Several limitations of this study must be considered. First, the absence of comprehensive assessments of quality of life and cognitive function limits the depth of our interpretative analysis. In future work, we plan to integrate a battery of standardized cognitive assessments to evaluate multiple domains of cognitive function in post-RT NPC patients and correlate them with neuroimaging findings for a better understanding of the interplay between radiation-induced changes in brain structure and function. Second, while key demographic and clinical variables such as age, sex, and tumor stage were well-matched, the lack of longitudinal data from the same individuals before and after RT is a significant limitation. This design introduces inter-individual variability, which may affect the robustness of our conclusions, so caution is needed when interpreting the results. Third, the relatively small sample size may have reduced statistical power, which should be taken into account when evaluating the findings. Fourth, as the study primarily focuses on neuroimaging, it does not include biomarker data from other areas, such as blood. To address these limitations, future longitudinal studies with larger sample sizes should incorporate a 2 × 2 factorial design to track changes in the same individuals over time. This approach would minimize the confounding effects of inter-individual variability and follow-up duration, providing more reliable and objective results to validate and expand upon our findings.

## Conclusion

Post-RT NPC patients exhibited reduced WW-FCS and GW-FCS in visual and motor coordination networks, with no WM functional alterations in temporal lobes, suggesting that radiation-induced WM dysfunction might precede within-field changes by occurring outside the radiation field.

## Data Availability

The raw data supporting the conclusions of this article will be made available by the authors, without undue reservation.
