# Supplementary material for: Radiation-induced white matter dysfunction in patients with nasopharyngeal carcinoma
Source: Front Neurosci. 2025 Mar 10;19:1548744. doi: 10.3389/fnins.2025.1548744 (PMC11931022; doi:10.3389/fnins.2025.1548744)
Supplement: Supplementary file 1 [file Data_Sheet_1.docx]

Table S1. Post-hoc power analysis of WW-FCS and GW-FCS differences between pre-RT and post-RT groups.

| Region | Sample Size | Cohen’s f^2^ | Power^*^ | Type II error |
| --- | --- | --- | --- | --- |
| *WW-FCS* |  |  |  |  |
| lh_Superior_cerebellar_peduncle | 111 | 0.084 | 0.86 | 0.14 |
| rh_Anterior_limb_of_internal_capsule | 111 | 0.096 | 0.90 | 0.10 |
| rh_Posterior_thalamic_radiation | 111 | 0.13 | 0.96 | 0.04 |
| lh_Posterior_thalamic_radiation | 111 | 0.10 | 0.92 | 0.08 |
| lh_Tapetum | 111 | 0.085 | 0.86 | 0.14 |
| *GW-FCS* |  |  |  |  |
| rh_Caudate | 111 | 0.10 | 0.92 | 0.08 |
| lh_Vis_4 | 111 | 0.10 | 0.91 | 0.09 |
| lh_Vis_5 | 111 | 0.092 | 0.89 | 0.11 |
| lh_Vis_8 | 111 | 0.11 | 0.93 | 0.07 |
| rh_Vis_4 | 111 | 0.093 | 0.89 | 0.11 |
| rh_Default_PFCv_2 | 111 | 0.10 | 0.92 | 0.08 |

* Power analysis was conducted using the WebPower package (Zhang & Yuan, 2018) in the R environment. Alpha level was set at 0.05.

Table S2. Post-hoc power analysis of partial correlation analysis between GW-FCS and MDRT.

| GW-FCS | MDRT | Sample Size | R-value | Power | Type II error |
| --- | --- | --- | --- | --- | --- |
| lh_Vis_8 | lh_Temporal | 37 | -0.46 | 0.81 | 0.19 |
| rh_Vis_4 | lh_Temporal | 37 | -0.38 | 0.63 | 0.37 |
| lh_Vis_8 | BrainStem | 37 | -0.35 | 0.55 | 0.45 |

References

Zhang, Z., & Yuan, K.-H. (2018). Practical Statistical Power Analysis Using Webpower and R (Eds). Granger, IN: ISDSA Press. https://doi.org/10.35566/power
